# Selection and Characterization of ssDNA Aptamers Targeting Largemouth Bass Virus Infected Cells With Antiviral Activities

**DOI:** 10.3389/fmicb.2021.785318

**Published:** 2021-12-17

**Authors:** Qing Yu, Mengmeng Li, Mingzhu Liu, Shuaishuai Huang, Gaoxue Wang, Taixia Wang, Pengfei Li

**Affiliations:** ^1^Guangxi Engineering Research Center for Fishery Major Diseases Control and Efficient Healthy Breeding Industrial Technology (GERCFT), Guangxi Academy of Sciences, Nanning, China; ^2^College of Life Science, Henan Normal University, Xinxiang, China; ^3^Guangxi Key Laboratory of Marine Natural Products and Combinatorial Biosynthesis Chemistry, Guangxi Beibu Gulf Marine Research Center, Guangxi Academy of Sciences, Nanning, China; ^4^Guangxi Key Laboratory of Beibu Gulf Marine Biodiversity Conservation, College of Marine Sciences, Beibu Gulf University, Qinzhou, China

**Keywords:** largemouth bass virus, aptamers, rapid detective technologies, antiviral effects, SELEX (Systematic Evolution of Ligands by Exponential Enrichment)

## Abstract

Largemouth bass virus (LMBV) is one of the most devastating viral pathogens in farmed Largemouth bass. Aptamers are novel molecule probes and have been widely applied in the field of efficient therapeutic and diagnostic agents development. LMBV-infected fathead minnow cells (LMBV-FHM) served as target cells in this study, and three DNA aptamers (LBVA1, LBVA2, and LBVA3) were generated against target cells by SELEX technology. The selected aptamers could specifically bind to LMBV-FHM cells, with rather high calculated dissociation constants (*K*d) of 890.09, 517.22, and 249.31 nM for aptamers LBVA1, LBVA2, and LBVA3, respectively. Three aptamers displayed efficient antiviral activities *in vitro*. It indicates that the selected aptamers have great potentials in developing efficient anti-viruses treatments. The targets of aptamers LBVA1, LBVA2, and LBVA3 could be membrane proteins on host cells. The targets of aptamers (LBVA1, LBVA2, and LBVA3) come out on the cells surface at 8, 10, 8 h post-infection. As novel molecular probes for accurate recognition, aptamer LBVA3 could detect LMBV infection *in vitro* and *in vivo*, it indicates that the selected aptamers could be applied in the development of rapid detective technologies, which are characterized by high sensitivity, accuracy, and easy operation.

## Introduction

Largemouth bass is commonly cultured worldwide, which has extremely economic and aquaculture significance (Wang et al., 2019). According to statistics, largemouth bass aquaculture production has exceeded 619,519 tons in 2020 in China (Zhang et al., 2021). However, the rapid development of largemouth bass aquaculture has led to frequent outbreaks of various pathogens. Among these pathogens, Largemouth bass virus (LMBV), a nucleoplasmic large DNA virus of the genus *Ranavirus* (family Iridoviridae), causes great economic losses and seriously threatens the largemouth bass breeding industry worldwide (Huang et al., 2014; Jia et al., 2020). There is an urgent need for research and development of rapid detection technology and efficient antiviral agents for preventing LMBV infection.

The key to control the outbreak of LMBV virus lies in the combination of active prevention and timely treatment (Li et al., 2021). The detective assays characterized of easy operation and high sensitivity are helpful to combat LMBV outbreaks in aquaculture. There have been several methods for LMBV diagnosis, such as polymerase chain reaction (PCR) assay (Iwanowicz et al., 2013), electron microscopy observation and analysis (Qi et al., 2016), loop-mediated isothermal amplification (LAMP) assay (Zhu et al., 2020), and so on. However, the application of PCR and electron microscopy assays in farms are limited by complex operations and expensive sophisticated equipment. Therefore, the development of novel practical detection assays for LMBV rapid diagnosis are urgently needed. Systematic evolution of ligands by exponential enrichment (SELEX) technology are able to generate artificial molecular probes, termed as aptamers (Phillips et al., 2008). Usually, aptamers are functional oligonucleotides (20–100 bases), which are generated from the randomly synthesized oligonucleotides library by SELEX (Yu et al., 2021b). Each aptamer has distinct nucleotide sequences, one of a kind, which enable each aptamer to form a distinct three-dimensional structure and specially bind to its target (Katilius et al., 2007). The target range of aptamers is very wide, including small molecules (Yang et al., 2016; Khoshbin et al., 2020), proteins (Zhou et al., 2016; Liang et al., 2017), virus particles (Hwang et al., 2012; Bai et al., 2018), bacteria (Yu et al., 2019a; Wang et al., 2020), cells (Zhang W. Y. et al., 2019; Zhou et al., 2020), and tissues (Liu et al., 2019; Lin et al., 2021). Aptamers are known as “artificial chemical antibodies” and have the advantages of strong specificity, high affinity and stability, easy chemical synthesis, and so on (Chong and Low, 2019). Based on aptamers’ excellent properties, aptamers are widely applied to develop rapid diagnosis technologies, which are characterized of easy operation and high sensitivity (Zhang Y. et al., 2019). For example, aptamer ST2P was generated targeting *Salmonella typhimurium*, a seriously harmful food borne pathogen. Aptamer ST2P was further used to develop the fluorescent bioassay for detecting *S. typhimurium*, whose detection limit was proved to be 25 cfu/mL (Duan et al., 2013).

In this study, DNA aptamers were generated against target LMBV-infected FHM cells by SELEX technology for the first time. The specificity, affinity, and antiviral ability of aptamers were systematically studied. The selected aptamers have great potentials in developing efficient anti-virus treatments and rapid detective technologies.

## Materials and Methods

### Ethics Statement

Procedures involving fish were performed in accordance with the ARRIVE (Animal Research: Reporting *in vivo* Experiments guidelines for reporting animal research), and approved by the Ethical Committee of the Guangxi Academy of Sciences (Nanning, China).

### Cell Lines and Virus Strains

Fathead minnow cells (FHM) and grouper spleen cells (GS) are used in this study (Qin et al., 2006). Largemouth bass virus (LMBV), grouper iridovirus (SGIV) (Xiao et al., 2019), soft-shelled turtle iridovirus (STIV) (Yu et al., 2021a), and grouper nervous necrosis virus (GNNV) (Yu et al., 2019b) were used to infect the cultured cells.

### Randomly Synthesized Oligonucleotides Library

Every oligonucleotide in a randomly synthesized library was composed of primer sequences at both ends and random sequences in the middle [5′-GACGCTTACTCAGGTGTGACTC G(50N)CGAAGGACGCAGATGAAGTCTC-3′]. The forward primer (FP) was labeled with 6-carboxy-fluorescein (FAM) (FAM-FP), and the reverse primer (RP) was labeled with biotin (Biotin-RP). The generated aptamers in this study were all labeled with FAM (FAM-aptamers). The synthesized library, FAM-FP, Biotin-RP, and FAM-aptamers were all synthesized by Aoke Biotech (Beijing, China).

### Systematic Evolution of Ligands by Exponential Enrichment Procedure

In this study, Virus Infected Fathead Minnow Cells (LMBV-FHM) were the target cells. FHM cells were seeded in culture dish (60 mm, *in vitro* scientific, Hangzhou, China) at 28°C for 24 h. FHM cells were infected with LMBV at a multiplicity of infection (MOI) of 1, 4 × 10^6^ LMBV-FHM cells were then collected for aptamers selection. The oligonucleotides library (10 nmol) was heated in water bath at 92°C for 5 min and incubated in ice for 10 min. The oligonucleotides library (10 nmol) was incubated with target LMBV-FHM cells for 60 min at 4°C. After being heated at 92°C for 3 min, the bound oligonucleotides were separated from the LMBV-FHM cells and used for PCR. The generated dsDNAs were firstly heated at 90°C for 2 min and then immediately put in ice. The ssDNAs mixtures containing FAM-forward ssDNAs and biotin-reverse ssDNAs were further incubated with streptavidin-labeled sepharose beads for 20 min at 4°C. FAM-ssDNAs were collected and served as oligonucleotides library for the next screening. For enhancing the specificity of oligonucleotides library for selection rounds, firstly, the number of LMBV-FHM cells decreased gradually (4.0 × 10^6^ for 1st round selection, 3.0 × 10^6^ for 2nd round, 2.5 × 10^6^ for 3rd round, 2.5 × 10^6^ for 4th round, 2.0 × 10^6^ for 5th round, 2.0 × 10^6^ for 6th round, 1.5 × 10^6^ for 7th round, 1.5 × 10^6^ for 8th round, 1.0 × 10^6^ for 9th round, 1.0 × 10^6^ for 10th round, 1.0 × 10^6^ for 11th round); secondly, incubation time of library and target cells gradually decreased (60 min for 1st round selection, 60 min for 2nd round, 50 min for 3rd round, 40 min for 4th round, 40 min for 5th round, 40 min for 6th round, 30 min for 7th round, 30 min for 8th round, 20 min for 9th round, 20 min for 10th round, 20 min for 11th round). From the 3rd round of selection, counter selection was also introduced. Briefly, ssDNA library generated in the previous round was incubated with normal FHM cells (4.0 × 10^6^) for 40–80 min. After centrifugation, the unbound oligonucleotides in supernatants were incubated with LMBV-FHM cells.

Flow cytometry (FACScan, Beckman Moflo XDP, German) was used to analyze the specific recognition of oligonucleotides library from different selection round. Briefly, the incubation of FAM-library (300 nmol/L) and LMBV-FHM cells served as control group (Control). Eleven rounds of screening were conducted, and the oligonucleotides library of 9th selection round were sequenced and analyzed by Aoke Biotech (Beijing, China). MFOLD software^1^ was further used to predict the secondary structures of selected aptamers.

### Identification of Selected Aptamers Specific Recognition for Largemouth Bass Virus-Fathead Minnow Cells by Fluorescence Observation Assay

The specific recognition of selected aptamers targeting LMBV-FHM cells was analyzed by fluorescence observation assay. Briefly, 1 × 10^4^ FHM cells were cultured in a glass bottom dish (Cellvis, catalog number D35-14-1-N). Cells were cultured at 28°C for 24 h, and then infected with LMBV (MOI = 1). At 24 h post-infection (hpi), LMBV-FHM cells were incubated with FAM-aptamers (300 nmol/L) at 4°C for 30 min. The cells in each dish were washed twice with PBS, and fluorescence observation was carried out by laser scanning confocal microscopy (LSCM, Nikon, Tokyo, Japan). The fluorescence observation of normal FHM cells incubated with FAM-aptamers (300 nmol/L) served as the control group.

### Identification of Selected Aptamers Specific Binding to Largemouth Bass Virus-Fathead Minnow Cells by Flow Cytometry Assay

The specific recognition of selected aptamers targeting LMBV-FHM cells was further analyzed by flow cytometry. FHM cells were infected with LMBV (MOI = 1). At 24 hpi, LMBV-FHM cells were incubated with FAM-aptamers (300 nmol/L) at 4°C for 30 min. The cells were washed twice with PBS, and fluorescence intensity detection was performed by flow cytometry (50,000 events). There were four control groups, Control1, fluorescence intensity detection of normal FHM cells incubated with FAM-aptamers (300 nmol/L); Control 2, fluorescence intensity detection of SGIV infected GS cells (SGIV-GS) incubated with FAM-aptamers (300 nmol/L); Control 3, fluorescence intensity detection of GNNV infected GS cells (GNNV-GS) incubated with FAM-aptamers (300 nmol/L); Control 4, fluorescence intensity detection of STIV infected FHM cells (STIV-FHM) incubated with FAM-aptamers (300 nmol/L). The gated areas were kept same for each group. The fluorescence intensity detection results are presented as the mean ± SD of three independent experiments.

### Characterization of Selected Aptamers Specificity *in vivo* by Flow Cytometry Assay

Healthy largemouth bass (approximately 10 g) were maintained in tanks supplied with the re-circulating water system for 10 days. Twenty fish were intraperitoneally injected with 50 μl LMBV (10^7^ TCID_50_/ml). There were two control groups: Con1, fish without LMBV treatments; Con2, fish were intraperitoneally injected with 50 μl PBS. Fish in different group were maintained in a separate tank with ample aeration. After 3 days, spleen tissues were collected from largemouth bass in different groups and were ground and diluted to 1 × 10^6^ cells in PBS. LMBV-infected fish cells were incubated with FAM-aptamers (300 nmol/L) at 4°C for 30 min. The cells were washed twice with PBS, and fluorescence intensity detection was performed by flow cytometry (50,000 events). The gated areas were kept same for each group. The fluorescence intensity detection results are presented as the mean ± SD of three independent experiments.

### Apparent Equilibrium Dissociation Constants Analysis

Dissociation constants of selected aptamers are carried out as reported previously (Li et al., 2015). Briefly, LMBV-FHM cells (1 × 10^6^) were incubated with FAM-aptamers at different concentrations (0, 62.5, 125, 250, 500, 1000 nmol/L) at 4°C for 30 min. LMBV-FHM cells were washed twice with PBS and collected for fluorescence intensity detection by flow cytometry (50,000 events). The fluorescence intensity detection of normal FHM cells incubated with FAM-aptamers at various concentrations (0, 62.5, 125, 250, 500, 1000 nmol/L) served as the control group. *K*d were calculated through the equation *Y* = BmaxX/(*K*d + X) by SigmaPlot. Bmax was the maximum fluorescence values of LMBV-FHM cells bound with FAM-aptamers. Y were the values of LMBV-FHM cells incubated with FAM-aptamers at various concentrations (X). *K*d of each aptamer is calculated as the mean ± SD of three independent experiments.

### Property Analysis of Aptamers’ Targets

Property analysis of aptamers’ targets on the LMBV-FHM cells surface were characterized by trypsin treatment assay. Briefly, LMBV-FHM cells were mixed in 500 μL trypsin (0.25%) for 5 min. The treated cells were incubated with FAM-aptamers (300 nmol/L) at 4°C for 30 min and washed twice with PBS, and then collected for fluorescence intensity detection by flow cytometry (50,000 events). Normal FHM cells incubated with FAM-aptamers (Control1), LMBV-FHM cells incubated with FAM-aptamers (Control2) served as two control groups.

The time points of aptamers’ targets appearing on the surface of LMBV-FHM cells were further characterized. Briefly, FHM cells were cultured at 28°C for 24 h, and then infected with LMBV (MOI = 1). At 0, 4, 6, 8, 10, 12, 14, 16 hpi, LMBV-FHM cells were incubated with FAM-aptamers (300 nmol/L) at 4°C for 30 min. The cells were washed twice with PBS and fluorescence intensity detection by flow cytometry (50,000 events).

### Aptamers’ Antivirus Activities

The antiviral activities of selected aptamers against LMBV infection *in vitro* were analyzed (Li et al., 2015). Briefly, 1 × 10^6^ FHM cells were cultured in 12-well plate at 28°C for 24 h. The mixtures of each aptamer (0.4 and 0.8 nmol) and LMBV (MOI = 0.5) were added into cells. LMBV (MOI = 0.5) alone added to cells (Con1), the random oligonucleotides library (0.8 nmol) mixed with LMBV (MOI = 0.5) added to cells (Con2) served as two control groups. At 24 hpi, the samples of cells and medium in each well were collected for quantitative reverse transcription PCR analysis (RT-qPCR) by quantifying the transcription of LMBV major capsid protein (*MCP*) gene. β*-actin* gene served as the internal control. The primers for detecting *MCP* and β*-actin* genes expression were shown in Table 1.

**TABLE 1:** The primers used for detecting LMBV infection in RT-qPCR.

Primers	Sequences
qMCP-F	5′-TGATTGGCAACACTAGCGATCT-3′
qMCP-R	5′-CCTAGCTCCTGCTTGATCGG-3′
β-actin F	5′-ATCGCCGCACTGGTTGTTGAC-3′
β-actin R	5′-CCTGTTGGCTTTGGGGTTC-3′

### Comparison of Largemouth Bass Virus Infection Diagnosis by Fathead Minnow Cells-Aptamer and Quantitative Reverse Transcription Polymerase Chain Reaction Analysis

Healthy largemouth bass (approximately 10 g) were maintained in tanks supplied with the re-circulating water system for 10 days. Twenty fish were intraperitoneally injected with 50 μl LMBV (10^7^ TCID_50_/ml). There were two control groups: Con1, fish without LMBV treatments; Con2, fish were intraperitoneally injected with 50 μl PBS. Fish in different group were maintained in separate tank with ample aeration. After 3 days, 100 mg spleen tissues from largemouth bass in different groups were ground and diluted to 5 × 10^3^ (Test 1), 1 × 10^4^ (Test 2), 5 × 10^4^ (Test 3), 1 × 10^5^ (Test 4), 5 × 10^5^ cells (Test 5)/sample in 200 μl PBS, respectively. The cells in each sample were collected for LMBV infection diagnosis by FAM-aptamer and RT-qPCR, respectively. Cells were incubated with FAM-aptamers (300 nmol/L) at 4°C for 30 min and washed twice with PBS, and then collected for fluorescence intensity detection by microplate reader instruments (Thermo). FAM-aptamer (300 nmol/L) incubated with 5 × 10^5^ of cells from fish without LMBV treatments, and 5 × 10^5^ of cells from fish intraperitoneally injected with PBS, served as the control groups, respectively.

### Statistical Analysis

All results are presented as the mean ± SD of three independent experiments. Intergroup differences were compared *via* one way analysis of variance by SPSS statistical software (IBM, Armonk, NY, United States). The results of comparisons with *P* < 0.01 are considered to represent statistically significant differences.

## Results

### Generation and Characterization of Aptamers Targeting Largemouth Bass Virus Infected Fathead Minnow Cells

**FIGURE 1:**
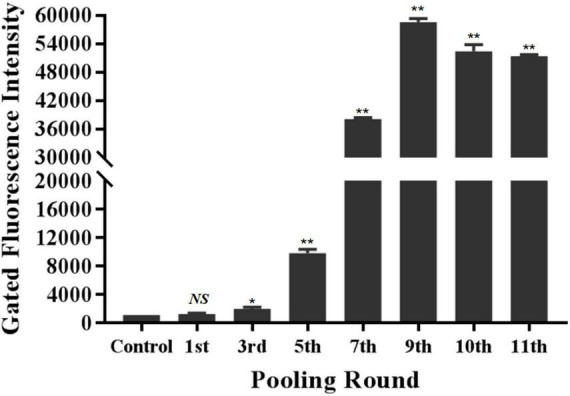
Generation and characterization of aptamers targeting LMBV-FHM cells. The specific recognition of oligonucleotides library from different selection round targeting LMBV-FHM cells was characterized by flow cytometry. With the increase of screening rounds, the fluorescence values of LMBV-FHM cells incubated with FAM-library from different selection round continuously increased. The fluorescence values peaked at the 9th round. The gated areas kept same for each group (*NS* means no statistical significance; *p < 0.05, **p < 0.01).

**FIGURE 2:**
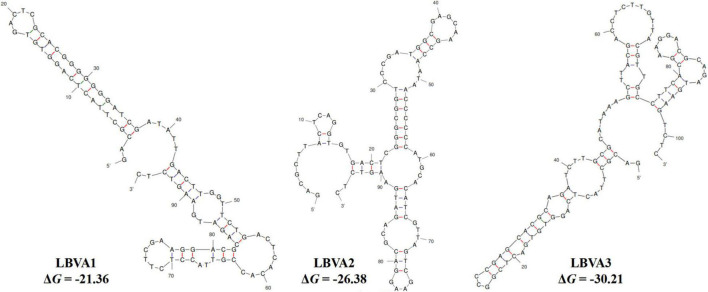
Secondary structures prediction of selected aptamers.

The specific recognition of oligonucleotides library from different selection round targeting LMBV-FHM cells was characterized by flow cytometry. As shown in Figure 1, with the increase of screening rounds, the fluorescence values of LMBV-FHM cells incubated with FAM-ssDNA library from different selection round continuously increased. The fluorescence values peaked at the 9th round, then the ssDNA in the 9th selection round pool were sequenced. Three aptamers LBVA1, LBVA2, and LBVA3 were obtained, which comprised 37, 30, and 24%, respectively (Supplementary Table 1). Aptamers’ sequences were shown in Table 2. As showed in Figure 2, there were similar stem-loop structures in the secondary structures of generated aptamers LBVA1, LBVA2, and LBVA3, and LBVA3 held lowest free energy (Δ*G*) value of −30.21 KJ/mol.

**TABLE 2:** Aptamers sequences.

Aptamer	Sequences
LMVA1	GACGCTTACTCAGGTGTGACTCGCACGGGGGGGATCGATATTGACTTGGTTCTGACTCACACCGTTACCTCTTCGAAGGACGCAGATGAAGTCTC
LMVA2	GACGCTTACTCAGGTGTGACTCGGGCGGTCCCGATGGCGAGCAAGCCAATAACCCCCCATGCACATCGTTAGTCGAAGGACGCAGATGAAGTCTC
LMVA3	GACGCTTACTCAGGTGTGACTCGGCCCGAGCACGCAGATCTTGCGCATAAAGCTTACGACCTCTTGTTACGTTGCCTTCACGAAGGACGCAGATGAAGTCTC

### Fluorescence Observation of Aptamers Specific Binding With Largemouth Bass Virus Infected Fathead Minnow Cells

**FIGURE 3:**
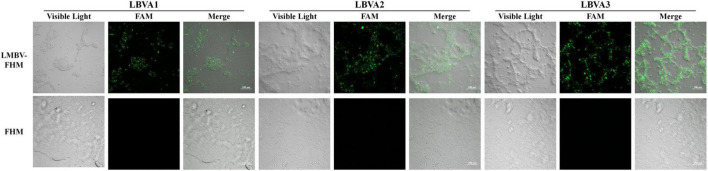
Fluorescence observation of aptamers specific binding with LMBV-FHM cells. There was significant fluorescence on the LMBV-FHM cell surface but little fluorescence around normal FHM cells in the control group.

The specific binding of aptamers LBVA1, LBVA2, and LBVA3 for target LMBV-FHM cells were identified by fluorescence observation. As shown in Figure 3, there were significant fluorescences on the LMBV-FHM cells surface, but there was little fluorescence around normal FHM cells in the control group.

### Specificity Characterization of Aptamers by Flow Cytometry

**FIGURE 4:**
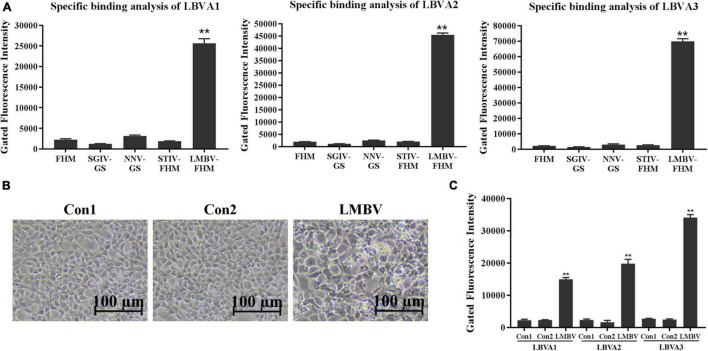
Specificity characterization of selected aptamers by flow cytometry. **(A)** Compared to low fluorescence values of the cells in control groups, there were obviously high fluorescence values on target LMBV-FHM cells incubated with FAM-LBVA1, FAM-LBVA2, and FAM-LBVA3, respectively. **(B)** Significant CPEs appeared in FHM cells incubated with filtrates from LMBV-infected fish tissues. **(C)** Compared to rather low fluorescence values on the fish spleen tissue cells in each control group, there were rather high fluorescence values on LMBV-infected fish cells incubated with FAM-LBVA1, FAM-LBVA2, and FAM-LBVA3, respectively. *NS* means no statistical significance; **p < 0.01.

The specific recognition of generated aptamers for LMBV-FHM cells was further evaluated by flow cytometry (Figure 4). Compared to rather low fluorescence values on the cells in each control group, there were obviously high fluorescence values on LMBV-FHM cells incubated with FAM-LBVA1, FAM-LBVA2, and FAM-LBVA3, respectively (Figure 4A). The results were consistent with the fluorescence observation results in Figure 3.

**FIGURE 5:**
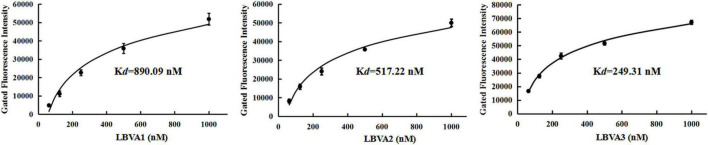
Dissociation constants analysis of selected aptamers. The calculated *K*_*d*_ for aptamers LBVA1, LBVA2, and LBVA3 were 890.09, 517.22, and 249.31 nM, respectively.

The spleen tissues harvested from largemouth bass were ground in PBS and centrifuged at 5,000 *g* for 10 min. The supernatants were filtered with a filter column (0.45 μm) and incubated with FHM cells at 28°C. After 48 h incubation, cells incubated with tissues filtrates from fish in Con 1 and Con 2 kept normal growth. By contrast, significant cytopathic effects (CPEs) appeared in cells incubated with filtrates from LMBV-infected fish tissues (Figure 4B). It indicated that fish intraperitoneally injected with virus were infected by LMBV. Compared to rather low fluorescence values on the fish cells in each control group, there were rather high fluorescence values on LMBV-infected fish spleen tissue cells incubated with FAM-LBVA1, FAM-LBVA2, and FAM-LBVA3, respectively (Figure 4C). It proved the specific recognition of aptamers LBVA1, LBVA2, and LBVA3 for the targets on LMBV-infected cells, but not to the uninfected ones. The *K*_*d*_ of aptamers LBVA1, LBVA2, and LBVA3 for target LMBV-FHM cells were 890.09, 517.22, and 249.31 nM, respectively (Figure 5).

### Property Characterization of Aptamers’ Targets

**FIGURE 6:**
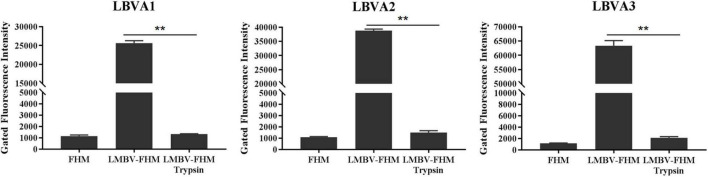
Property characterization of aptamers’ targets. Compared to rather high fluorescence values on the LMBV-FHM cells incubated with each FAM-aptamer (LBVA1, LBVA2, and LBVA3), the fluorescence values on trypsin-treated LMBV-FHM cells decreased obviously (**p < 0.01).

The property analysis of aptamers’ targets for LMBV-FHM cells were also evaluated by flow cytometry. Compared to rather high fluorescence values on the LMBV-FHM cells incubated with each FAM-aptamer (LBVA1, LBVA2, and LBVA3), the fluorescence values on trypsin-treated LMBV-FHM cells decreased obviously (Figure 6). It indicated that trypsin treatments could remove the aptamers’ binding targets in LMBV-FHM cells.

**FIGURE 7:**
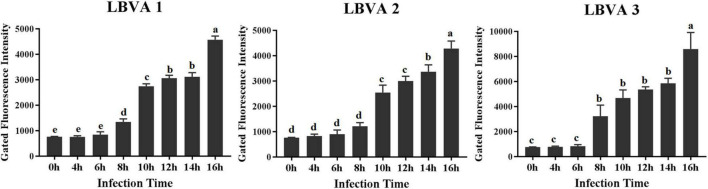
Appearance time identification of aptamers’ target proteins on LMBV-FHM cells surface. Obviously increasing fluorescence values on LMBV-FHM cells appeared at 8, 10, and 8 hpi for FAM-LBVA1, FAM-LBVA2, and FAM-LBVA3, respectively (bars with different superscript are significantly different, *p* < 0.01).

The appearance time of aptamers’ target proteins on LMBV-FHM cells surface was identified by flow cytometry analysis. As shown in Figure 7, after incubation with FAM-aptamers, there were obviously increasing fluorescence values on LMBV-FHM cells incubated with FAM-LBVA1, FAM-LBVA2, and FAM-LBVA3 at 8, 10, and 8 hpi, respectively.

### Antiviral Activities of Aptamers Against Largemouth Bass Virus Infection *in vitro*

**FIGURE 8:**
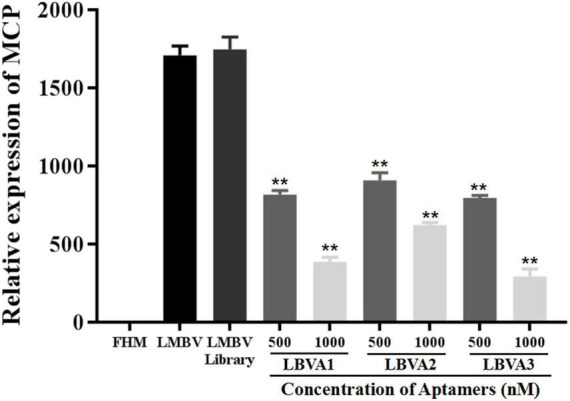
Antiviral activities of aptamers against LMBV infection *in vitro*. Compared to the control groups, the relative mRNA expression of LMBV *MCP* gene in FHM cells treated with both LMBV and aptamers (LBVA1, LBVA2, and LBVA3) decreased significantly at 24 hpi (**p < 0.01).

The antiviral abilities of selected aptamers LBVA1, LBVA2, and LBVA3 were identified by quantifying the transcription of LMBV *MCP* gene *via* RT-qPCR. As shown in Figure 8, the random oligonucleotides library exhibited no anti-LMBV effects. By contrast, LMBV *MCP* gene expression in cells treated with LMBV and aptamers (LBVA1, LBVA2, LBVA3) decreased obviously (Figure 8).

### Comparison of Largemouth Bass Virus Infection Diagnosis by FAM-LBVA3 and Quantitative Reverse Transcription Polymerase Chain Reaction Analysis

**FIGURE 9:**
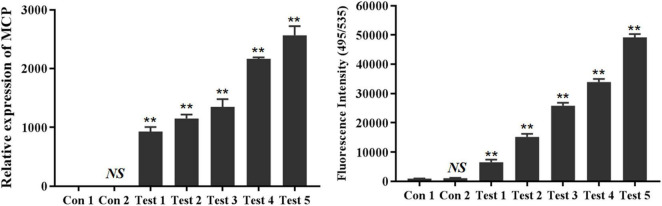
FAM-LBVA3 applied for rapid diagnosis of LMBV infection. Groups of fish without LMBV treatments, and fish intraperitoneally injected with PBS, served as two control groups. Compared to the control groups, both RT-qPCR (Left) and FAM-LBVA3 (Right) could identify LMBV infection in virus-infected fish spleen cells at 5 × 10^3^, 1 × 10^4^, 5 × 10^4^, 1 × 10^5^, 5 × 10^5^ cells/sample, respectively (**p < 0.01).

Both FAM-labeled aptamer LBVA3 (FAM-LBVA3) and RT-qPCR could identify LMBV infection in virus-infected fish tissues cells at 5 × 10^3^, 1 × 10^4^, 5 × 10^4^, 1 × 10^5^, 5 × 10^5^ cells/sample, respectively (Figure 9). Aptamer LBVA3 detection results were consistent with RT-qPCR results.

## Discussion

Aquaculture diseases that result from virus infection are the major cause of high mortality, and it is necessary to develop effective approaches of rapid and reliable diagnostics and treatments. The unique nucleotide sequence of each aptamer forms the basis of each aptamer’s distinct complex three-dimensional structure, which further makes aptamer recognize the target with rather high specificity (Hasegawa et al., 2016). In past decades, aptamer technology developed rapidly in various scientific fields (Tan et al., 2013; Yu et al., 2021b). Hundreds of aptamers targeting virus proteins, virus particles, and virus-infected cells have been generated and widely applied to develop detective technologies and efficient antiviral treatments (Chong and Low, 2019). In this study, ssDNA aptamers (LBVA1, LBVA2, and LBVA3) targeting LMBV-infected cells were generated and characterized for the first time.

The diagnostic technologies characterized of high sensitivity and easy operation are useful to control aquatic pathogens outbreaks. Nowadays, PCR and immunological methods are broadly applied. PCR assay is based on the analysis of pathogens’ DNA/RNA fragments. PCR is the most specific and sensitive detection assay and could be used to identify various pathogens. Immunological methods are based on the specific recognition of antibodies targeting pathogens’ unique glycoproteins, glycolipids, and proteins. However, PCR operation is a rather complex technology that requires expensive reagents, instruments, and professional technicians. The antibodies used in the immunological methods need rather strict storage conditions to avoid antibodies inactivation. Therefore, the need for research on cost-effective and practical pathogens detection assays remain urgent (Yu et al., 2021b).

In the past decade, aptamers targeting pathogens have attracted more and more attention. As novel molecular probes for accurate recognition, aptamers have been widely applied to develop detective technologies, and they are always characterized with chemical stability, high sensitivity, accuracy, and easy operation (Duan et al., 2016). For example, ssDNA aptamer ST2P was generated against *S. typhimurium via* whole-bacterium-based SELEX. Aptamer ST2P-based fluorescent bioassay was further established and applied to detect *S. typhimurium*, whose limit of detection was as low as 25 cfu/mL (Duan et al., 2013). Aptamer Q3 was selected targeting SGIV-infected cells, which could potentially serve as the molecule probe in diagnostic assay targeting SGIV infection. Aptamer Q3 was first applied in the development of enzyme-linked apta-sorbent assay (ELASA) for detecting SGIV infection *in vitro* and *in vivo*, which were characterized with high stability, sensitivity, and accuracy (Li et al., 2016). In this study, a LBVA3-based fluorescent molecular probe was successfully applied to identifying LMBV infection. The complete detection process took less than 1 h, with rather easy operations. It is worth noting that the accuracy of aptamer LBVA3-based detection results was consistent with RT-qPCR. Then aptamer LBVA3 had great potentials in development of novel detection assay.

Another pressing challenge is developing efficient anti-virus treatments. In some cases, aptamers targeting virus displayed antiviral activities by blocking the functions of target proteins (Hwang et al., 2012; Woo et al., 2013; Gao et al., 2014; González et al., 2016; Liang et al., 2017). For example, human immunodeficiency virus (HIV) is responsible for 3 million deaths every year, and there is an urgent need to obtain novel antiretroviral agents. Reverse transcriptase of HIV plays a key role during virus replication, and lots of aptamers have been generated targeting reverse transcriptase of HIV. Some selected aptamers could inhibit the HIV replication *in vitro* (Joshi et al., 2005; Lange et al., 2012). HIV gp120 is another promising target for generating anti-virus aptamers. Aptamer B40 exhibited antiviral effects by blocking the conservative amino acids in the region of gp120 binding with CCR5 (Cohen et al., 2008; Joubert et al., 2010). Hemagglutinin (HA) of influenza virus holds a key role in starting virus infection by binding to the receptors and further mediating virus entry into host cells. Thereby, HA is a potential target for developing aptamer-based anti-influenza agents. Two DNA aptamers, A21 and A22, were generated targeting HA-(91-261) peptide, which is the highly conservative region of HA. The aptamers could fight against influenza virus infection both *in vitro* and *in vivo* by blocking virus binding to cell receptor and virus invading into the host cells (Jeon et al., 2004). Chen et al. (2009) generated DNA aptamer ZE2 against envelope E2 protein of Hepatitis C virus (HCV). Aptamer ZE2 could specially bind to HCV virions and block virus fusion with cells. The results indicate that aptamer ZE2 have great potentials in the field of therapeutic and diagnostic agents development. RNA aptamers (H1, H2, H3, and H4) were selected against hirame rhabdovirus (HIRRV). As these aptamers could combat HIRRV infection in the dose-dependent way, they could serve as powerful tools for the control of HIRRV infection (Hwang et al., 2012). Grass carp reovirus (GCRV) is a seriously harmful pathogen, which results in rather high mortality rate of grass carp aquaculture. Aptamers targeting GCRV S10 protein could obviously inhibit the virus infection. When aptamers incubated with cells for 1 h before GCRV addition, aptamers showed the most significant inhibitory effects. The selected aptamers could exhibit the anti-GCRV effects by interfering with virus binding to host cell surface (Liang et al., 2017). The aptamers described above are all targeting proteins or virus particles, and aptamers targeting virus-infected host cells are of great interest. The targets of reported aptamers against cells are mainly cell membrane proteins (Yu et al., 2021b). As is known to all, membrane proteins have various functions, and the research on membrane proteins changes greatly helps explore the disease pathogenesis and develop effective treatments (Seeger and Mason, 2015; Yu et al., 2020). Trypsin could degrade membrane proteins of cultured cells (Yu et al., 2021a). In this study, trypsin treatments made the binding between selected aptamers (LBVA1, LBVA2, and LBVA3) and LMBV-FHM cells disappear. It indicates that three aptamers’ targets are cell membranes. In order to identify and characterize the targets of aptamers (LBVA1, LBVA2, LBVA3) in LMBV-infected cell membranes, LMBV-infected cells at different time points were incubated with FAM-aptamers (LBVA1, LBVA2, LBVA3) and fluorescence intensities of each group were analyzed by flow cytometry. As shown in Figure 7, for LBVA1 and LBVA3, fluorescence intensities in the groups of LMBV-FHM cells at 6 and 8 hpi were significantly different (**P < 0.01); furthermore, fluorescence intensities in the groups of LMBV-FHM cells at 0, 4, and 6 hpi were not statistically significant. For LBVA2, fluorescence intensities in the groups of LMBV-FHM cells at 8 hpi and 10 hpi were significantly different (^**^P < 0.01); furthermore, fluorescence intensities in the groups of LMBV-FHM cells at 0, 4, 6, and 8 hpi were not statistically significant. It indicates that the appearance time of aptamers’ target proteins on LMBV-FHM cells surface are 8, 10, and 8 hpi for LBVA1, LBVA2, and LBVA3, respectively. The anti-LMBV analysis results of the aptamers showed that all aptamers (LBVA1, LBVA2, and LBVA3) could combat LMBV infection *in vitro*, and LBVA3 had the best antiviral ability. Then aptamer LBVA3 could be applied in antiviral agent development. The detailed mechanisms of aptamer LBVA3 inhibiting LMBV infection requires more exploration in future studies. Furthermore, it is necessary to isolate and characterize the targets of aptamers (LBVA1, LBVA2, LBVA3), which could help explore LMBV pathogenesis.

## Conclusion

Three DNA aptamers (LBVA1, LBVA2, and LBVA3) were selected against target LMBV-FHM cells by SELEX technology. The selected aptamers could specifically bind to LMBV-FHM cells. Three aptamers displayed efficient antiviral activities *in vitro*. The targets of aptamers could be membrane proteins on host cells. Aptamers could detect LMBV infection *in vitro* and *in vivo*, and it indicates their great potentials in rapid detective technologies development.

## Data Availability Statement

The original contributions presented in the study are included in the article/Supplementary Material, further inquiries can be directed to the corresponding author.

## Ethics Statement

The animal study was reviewed and approved by Ethical Committee of the Guangxi Academy of Sciences.

## Author Contributions

QY performed the main experiments and helped to prepare the manuscript. MeL performed the flow cytometry analysis. MiL helped to perform the LSCM experiments and analysis tools. SH contributed to the reagents, materials, and experimental cells. GW helped to design the experiments. TW helped to prepare the manuscript. PL conceived and designed the experiments and prepared the manuscript.

## Conflict of Interest

The authors declare that the research was conducted in the absence of any commercial or financial relationships that could be construed as a potential conflict of interest.

## Publisher’s Note

All claims expressed in this article are solely those of the authors and do not necessarily represent those of their affiliated organizations, or those of the publisher, the editors and the reviewers. Any product that may be evaluated in this article, or claim that may be made by its manufacturer, is not guaranteed or endorsed by the publisher.

## Footnotes

1
https://sfold.wadsworth.org/cgi-bin/srna.pl
