# Supplementary material for: Selection and Characterization of ssDNA Aptamers Targeting Largemouth Bass Virus Infected Cells With Antiviral Activities
Source: Front Microbiol. 2021 Dec 17;12:785318. doi: 10.3389/fmicb.2021.785318 (PMC8718865; doi:10.3389/fmicb.2021.785318)
Supplement: Supplementary file 1 [file Table_1.DOC]

**Table S1** Identification of ssDNA aptamers targeting LMBV-infected FHM cells

| Apatamer | Central randomized sequences | Frequences(%) |
| --- | --- | --- |
| LBVA1 | GACGCTTACTCAGGTGTGACTCGCACGGGGGGGATCGATATTGACTTGGTTCTGACTCACACCGTTACCTCTTCGAAGGACGCAGATGAAGTCTC | 37 |
| LBVA2 | GACGCTTACTCAGGTGTGACTCGGGCGGTCCCGATGGCGAGCAAGCCAATAACCCCCCATGCACATCGTTAGTCGAAGGACGCAGATGAAGTCTC | 30 |
| LBVA3 | GACGCTTACTCAGGTGTGACTCGGCCCGAGCACGCAGATCTTGCGCATAAAGCTTACGACCTCTTGTTACGTTGCCTTCACGAAGGACGCAGATGAAGTCTC | 24 |
| LBVA4 | GACGCTTACTCAGGTGTGACTCGTGTTCGCCAGACGTGTGCCAGATTCCCACAGGTTGGATGCCAGTTGTGCTCGAAGGACGCAGATGAAGTCTC | 1 |
| LBVA5 | GACGCTTACTCAGGTGTGACTCGTGGCACGCCACAACGAAGGGCATAGTTCAGCCCCAATACCAACGTCGCTTCGAAGGACGCAGATGAAGTCTC | 1 |
| LBVA6 | GACGCTTACTCAGGTGTGACTCGTTGCACCGCATCGTCACGGGCACTCGAGGCAAATTCGGACACCAAACCGTCGAAGGACGCAGATGAAGTCTC | 1 |
| LBVA7 | GACGCTTACTCAGGTGTGACTCGCATCATTGAACGCTAACCCCACTCGTTTCCTCGATCCAGCATTTCACCTTCGAAGGACGCAGATGAAGTCTC | 1 |
| LBVA8 | GACGCTTACTCAGGTGTGACTCGCTATGCCTACTGTTTTGTTTCAGAAGGGTCGAACACACGGATAGCTCCATCGAAGGACGCAGATGAAGTCTC | 1 |
| LBVA9 | GACGCTTACTCAGGTGTGACTCGCCACCTCAAACGATACCGGTCGCAATTCTCACCACTCGCTCTACACCACACGAAGGACGCAGATGAAGTCTC | 1 |
| LBVA10 | GACGCTTACTCAGGTGTGACTCGACCGAAGTGTATAAGTGATAAGACGAACGACGGGGGGTTCAACATGCCCTCGAAGGACGCAGATGAAGTCTC | 1 |
| LBVA11 | GACGCTTACTCAGGTGTGACTCGTGCCCAGCGTCTGCCAGTGTTTCCTGGTGGCCCGACCATATTCTCCAAATCGAAGGACGCAGATGAAGTCTC | 1 |
| LBVA12 | GACGCTTACTCAGGTGTGACTCGGTGTCGTACCGTTGTGTCGCGCGGGCAAGGGCGTGTCTGCTGACTGACCCGAAGGACGCAGATGAAGTCTC | 1 |

Frequency indicates percentage of the aptamer candidates in the 9^th^ selected pool.
